# Challenges associated with coronavirus disease (COVID-19)-related self-quarantine in Ghana: lessons for future self-quarantine interventions

**DOI:** 10.11604/pamj.2024.47.5.41064

**Published:** 2024-01-05

**Authors:** Anaman Stephen, Mbuyiselo Douglas, Frederick Ngmenkpieo, Gregory Kofi Amenuvegbe, Prince Owusu Adoma, Manu Emmanuel

**Affiliations:** 1 Department of Population and Behavioural Sciences, Fred N. Binka School of Public Health, University of Health and Allied Sciences, Hohoe, Ghana,; 2 Department of Public Health, Faculty of Health Sciences, Walter Sisulu University, Private Bag X1, Mthatha 5117, South Africa,; 3 Department of Educational Foundations, School of Education and Lifelong Learning, SD Dombo University of Business and Integrated Development Studies, Wa, Ghana,; 4 Department of Health Policy Planning and Management, Fred N. Binka School of Public Health, University of Health and Allied Sciences, Hohoe, Ghana,; 5 Department of Health Administration and Education, Faculty of Science Education, University of Education, Winneba, Ghana

**Keywords:** COVID-19 prevention, quarantine, Eastern Region, infectious disease prevention, Ghana Health Service

## Abstract

**Introduction:**

self-quarantine was one of the key public health interventions in halting the spread of the coronavirus disease (COVID-19) in Ghana. Despite its success, self-quarantine was bridled with challenges across the country, including in the Eastern Region. Consequently, it was pertinent to ascertain these challenges to inform future self-quarantine interventions in the region and the country. The study aimed to ascertain challenges faced by COVID-19 self-quarantined persons in the Eastern Region of Ghana to inform future policies on self-quarantine in the region and the country in general.

**Methods:**

thirty-five (35) participants were interviewed in both Twi and English. Following the thematic content analysis approach, Atlas. ti software was used to analyse the data. Relevant quotes were extracted from the transcripts to back the various sub-themes in presenting the results.

**Results:**

three global themes emerged from the analyses: socio-economic challenges of self-quarantine (lack of access to essential goods and services, loss of income, and poor housing conditions), health-related challenges (sedentary lifestyle, non-supply of essential personal protective equipment such as face masks, development of oedema and weight gain), and psychological challenges (loneliness, boredom, and anxiety).

**Conclusion:**

COVID-19-related challenges self-quarantined persons faced in the Eastern Region of Ghana were multifaceted, ranging from socio-economic, and health to psychological ones. Consequently, emergency preparedness for future pandemic control using self-quarantine as a tool should bring on board various stakeholders to ensure challenges identified in this study are holistically addressed and do not recur.

## Introduction

Worldwide, several public health interventions were implemented during the coronavirus disease (COVID-19) outbreak to halt the spread of the disease [1]. Ghana adopted quarantine as one of its key public health interventions to halt the spread of COVID-19. This approach was in two forms: mandatory quarantine and self-quarantine. Individuals entering the country through approved borders were quarantined in hotels, as a form of mandatory quarantine, while those within the country who had been suspected of encountering infected COVID-19 persons but were not showing signs of the disease, were made to undergo self-quarantine in their homes for the incubation period of the disease [2]. Irrespective of whether mandatory or self-quarantine, suspected individuals were separated from the general population for the incubation period of the virus and under close monitoring until such individuals were cleared of the disease [3]. While the ethics and legality of quarantine have been questioned in the past [4], it remains a necessary public health intervention for preventing the spread of infectious diseases, as it facilitates early detection of the disease in question, for rapid implementation of response measures [5]. Despite the positive attribute of quarantine in public health practice, it has been found to have some negative health consequences on quarantined persons. For instance, quarantine has been found to reduce indulgence in physical activity for those concerned and alter their food choices, leading to cardiovascular diseases [6]. It is also an unfriendly experience for those who are involved, due to separation from loved ones, loss of freedom, uncertainty over disease status, and boredom [7]. Anecdotal evidence from the Eastern Regional Health Directorate of Ghana indicates that the region recorded its first case of COVID-19 in the Lower Manya Krobo Municipality on March 31, 2020, which was an imported case from India. The infected person later had contacts in the district, which then extended to other districts in the region. By the end of August 2020, all regional districts had recorded COVID-19 cases, with a cumulative case count of 2,285. As a result, contact tracing and subsequent quarantine of suspected contacts of infected persons were instituted.

However, due to the limited resources of the Regional Health Directorate and the government, public isolation centres could not be set up within the Eastern region to enforce holistic mandatory quarantine [8]. As a result, suspected individuals were asked to undergo self-quarantine in their homes. While mandatory quarantine was a bit comfortable for persons involved because they were accommodated in hotels that were equipped with basic amenities such as toilets, and provided with nutritious meals, the same cannot be said about self-quarantine. With the latter, individuals had to be isolated in their homes or rooms, irrespective of the availability of basic amenities such as running water and private toilets. This approach was daunting for affected individuals who underwent self-quarantine [9]. Although it is believed that persons who underwent self-quarantine in Ghana and in the Eastern region, in particular, faced some challenges, these challenges have not been investigated and reported to inform future self-quarantine practice in the region and the country as a whole. Moreover, the literature on COVID-19 prevention in Ghana, in general, is skewed toward compliance with prevention protocols such as social distancing, handwashing, and wearing face masks [10-14]. Thus, evidence of key public health interventions that played a key role in halting the spread of the disease, such as self-quarantine, is limited, including in the Eastern region. However, the few studies in this area of disease prevention only delved into the challenges of contact tracing [15,16] but not the challenges individuals who underwent self-quarantine because of contact tracing faced. We, therefore, ascertained the self-reported challenges of contacts who underwent self-quarantine faced in the Eastern Region of Ghana to help better inform future self-quarantine interventions in the region and the country as a whole.

## Methods

### Study site and period

The study was conducted in the Eastern Region of Ghana in July 2021. The region lies between latitudes 6 and 7 degrees North and longitude 1.30 West and 0.30 degrees East. It is the sixth largest region with a land area of 19,323 kilometres square, which is 8.1% of the land area of Ghana [17]. The region shares boundaries with five other regions, namely the Greater Accra, Volta, Bono East, Ashanti, and Central regions. Its capital is Koforidua, which is located in the New Juaben Municipality. The region was chosen for a case study to ascertain the general challenges that self-quarantined persons faced at the height of the COVID-19 pandemic in Ghana.

### Study design

The phenomenological qualitative research design was deployed to ascertain the lived experiences of individuals who underwent COVID-19-related self-quarantine in the Eastern Region of Ghana. We deployed this design as we aimed to ascertain the lived experiences of participants in relation to challenges they faced during the COVID-19 pandemic, on the assumption that participants would be willing to share their lived experiences during self-quarantine.

### Study participants and recruitment process

Thirty-five (35) individuals who had undergone COVID-19-related self-quarantine in the Eastern Region of Ghana were interviewed. To be included in the study, participants had to be at least eighteen (18) years of age and had to have undergone the mandatory two-week self-quarantine process within the Eastern Region. Potential participants who met the inclusion criteria, but refused to cooperate or were indisposed at the time of the study were excluded. The sampling procedure was purposive, as only individuals who met the inclusion criteria and were ready to participate were recruited into the study. Potential participants were contacted through the Regional Disease Control Officer, via the various District Disease Control Officers.

### Data collection procedure

A semi-structured in-depth interview guide was used to collect the data. The guide consisted of questions on the socio-demographic characteristics of participants, as well as challenges participants faced during self-quarantine. The interviews were conducted in secluded locations suggested by the participants within their communities and their homes. Each participant was assigned a numeric code before the interview so as not to divulge their personal identities during the interview and data analysis processes, as the codes helped to track the source of each interview transcript. Depending on the language preference of the participants, interviews were either conducted in English or Twi. Seven interviews were conducted in English and the rest in Twi. The interviews were conducted by the Principal Investigator, who is a Public Health Promotion Officer, under the supervision of the team leader, with each lasting about an hour. They were recorded with an Olympus audio recorder, with the permission of participants.

### Ethical considerations

Ethical approval for this study was sought from the University of Health Allied Sciences Research Ethics Committee (UHAS-REC A.10 [69] 20-21). Permission was obtained from the Eastern Regional Health Directorate. Informed consent was sought from the participants by fully disclosing the content of the study to them. Participants gave their consent by signing or thumbprinting an informed consent form after its content had been thoroughly explained to them.

### Data analysis

The transcripts were transcribed by a qualified translator vexed in the English and Twi languages. The interviews conducted in the Twi language were directly transcribed into English and then handed to the Principal Investigator. The transcripts were then duplicated and shared between two independent coders, who followed the inductive content analysis approach, as we did not have preconceived themes before the analysis [18]. The transcripts were loaded into the ATLAS.ti version 9.0 software for analysis, after they had been thoroughly read and the content familiar. Colour coding was done by the two independent coders for theme formation and categorisation. After the initial codes were developed by the two independent coders, the team, led and the Principal Investigator, had a meeting with the two coders to review the codes. During the meeting, codes that were similar in meaning were merged in terms of agreement on the most expressive codes/phrases as the sub-theme for those codes. However, codes with distinct meanings were treated as independent sub-themes. The sub-themes were then grouped under various themes. Relevant quotes were then extracted from the transcripts to support each sub-theme in presenting the data.

### Quality control processes

To ensure participants provided us with credible responses, before the interviews, we created rapport with potential participants for them not to feel stigmatised. This ensured they felt comfortable enough to share their experiences with us. After the transcription process was completed, The Principal Investigator reverted to some of the participants who could be reached with the transcripts to confirm that our translations and transcriptions were true reflections of what they said during the interviews before analyses were carried out. Two peer qualitative researchers were tasked to review the transcripts to authenticate the translations before the data were analysed. Furthermore, we documented and described our methods in detail to ensure that the study could be replicated should the need arise. The interviewer also eschewed preconceived ideas during the interviews to minimise bias. Lastly, each participant was given the laxity to freely express their views without interference from the interviewers.

## Results

Thirty-five (35) participants were involved in the study, comprising 15 males and 20 females. The ages of the participants ranged from 18 to 60 years. Only 7 out of the 35 had a tertiary level of education. Twenty-one (21) were privately employed, 8 were civil servants and 6 were unemployed. Out of the 35, 21 were married, 26 of them were Christians and the rest were Muslims. Three global themes emerged from the analyses, namely: socioeconomic challenges of self-quarantine, health-related challenges, and psychological challenges associated with self-quarantine.

### Challenges encountered during self-quarantine

Under this global theme, three themes emerged; socio-economic challenges associated with self-quarantine, health challenges, and psychological challenges.

### Socio-economic challenges

Participants mentioned three socio-economic challenges that they faced under self-quarantine. Lack of access to essential goods and services, loss of income, and poor housing conditions were the challenges participants alluded to having encountered when placed under self-quarantine.

#### Lack of access to essential goods and services

Almost all the participants lamented that they had difficulty accessing essential services, including not having access to essential services such as food and medications, as their movements were restricted. This was the case for individuals who lived alone, as self-quarantine did not permit them to go about their normal daily routines. A participant explained: *“I develop some headaches in the quarantine but the person who was taking care of me I wanted the person to get medicine for me from the drug store, but unfortunately for me the person who was taken care me I try to get in touch with that she can get some pain kills for me at the drugs store. I learned she has travelled and where she had gone to, there was no good telecommunication network so, I could not reach her. So, I suffered a lot with the headache [Male, 40 years old]”*.

### Loss of income

Fourteen (14) of the participants expressed concern about the economic hardship they had to endure during the period of the quarantine. They either had poor sales, as were not physically around to attend to their customers, had their businesses monetarily halted, or had their businesses collapse entirely because of stigmatisation by customers. Verbatim quotation from one of them illustrates this point: *“Mmm! You know that in the taxi business, we were taking only three passengers [during COVID-19], so I had no money by then [when told to self-quarantine]. The money I give to the family for upkeep even decreased and a lot of pressure was on me. In fact, things became hard for me, and I am yet to recover” [Male, 28 years old]*.

#### Poor housing conditions

Most of the participants also faced poor housing conditions during self-quarantine. Either they had to wait for long hours or waited late into the night before they could access washrooms, as they lived in homes where these facilities were shared. Some also struggled with access to water, as they had no water in their homes and had to rely on public water sources. These conditions made it difficult for them to effectively self-quarantine. Although they were not supposed to interact with the public, some ended up sharing bathrooms with co-tenants in their homes. A participant explained as follows: *“Our house is the old type of self-contained house whereby we have a fence but use a common washroom and bathhouse. so, with that, because I was under quarantine, I needed to wait for people to take their baths before I could do the same. In the evening I was the last person to bathe. You can imagine if nature calls you and someone is there, it was a challenge” [Male, 35 years old]*.

#### Health-related challenges

Participants also mentioned some health-related challenges they faced during self-quarantine. These included a sedentary lifestyle, inadequacy of personal protective equipment that could have exposed others to the disease, swollen feet, and obesity.

#### Sedentary lifestyle

Three participants explained how self-quarantine altered their daily exercise and confined them to their rooms without much physical activity. They were, thus, forced into a sedentary lifestyle, which they believe, affected their health. One of them narrated: *“I am an athlete so a jog every morning and evening, at least for three kilometres, but when I was quarantined, I could not do that. I was only eating and going to bed, aside from a few exercises I was doing in the room which cannot be compared to jogging. I was actually not prepared for it (self-quarantine)” [Male, 38 years old]*.

#### Non-supply of essential personal protective equipment (face masks)

As a public health requirement, individuals under self-quarantine were always supposed to be in face masks. Since not everyone could afford these masks, and as a public health intervention, self-quarantined individuals were supposed to be supplied with this essential personal protective equipment. However, participants faced a shortage of these essentials, exposing their caregivers to the virus. One of them narrated as follows: *“When they [contact tracers] asked me to [self] quarantine, I asked them whether they will be feeding me, but they said no. That they will only give me face masks to protect myself and other people. When they came to take my sputum [sample] the following day, I asked them about the masks they promised, and they said they will bring them. I only saw them again in two weeks´ time when they came to tell me that I am free to leave the room now” [Female, 55 years old]*.

#### Oedema

Two of the participants complained that they developed oedema because of physical inactivity during self-quarantine. Lack of movement made their feet swell and they attributed this to being under self-quarantine. One explained: *“I always had swollen feet. I even called them [contact tracers] and complained about that but they only told me to be exercising in the room. Maybe it was because I was always sitting on the bed watching television. But now, I am okay” [Female, 42 years old]*.

#### Weight gain

Some participants indicated that they gained extra weight during self-quarantine. One participant explained that she easily gains weight and to control that, she exercises regularly through jogging and going to the gymnasium. However, as a result of self-quarantine, she could not keep up with her fitness regime and gained extra weight in the process. Her view on the negative effects of self-quarantine were expressed as follows: *“I wish you knew me before I was [self] quarantined. I have lost my shape as I have gained more weight since I returned from [self] quarantine. My Boss [at work] could not even recognize me when he saw me. Some of us need to be walking freely in order not to gain much weight. It [gaining weight quickly] is in the family so once you eat and don't exercise, you are likely to become fat” [Female, 34 years old]*.

#### Psychological challenges

Under this theme, three challenges were mentioned by participants: having faced loneliness, boredom, and anxiety.

#### Loneliness

Most of the participants mentioned that they felt lonely while under self-quarantine, as their only companion was the television. Thus, they missed the normal interactions they used to have with their families and wished they could return to their normal lifestyles. A male explained this: *“How can you stay alone in a room without being lonely? I am a married man, but I was forced to abandon my family and confine myself in a small room. The children missed me, and I also missed them, but I had to do it for the good of all of us. It is not a nice experience” [Male, 45 years old]*.

#### Boredom

Being alone, with an accustomed daily routine, which was to eat, watch television, and sleep, some participants mentioned that they became bored in their rooms. To kill the boredom, some resorted to reading and listening to music. One participant narrated: *“hmmm, I will not even wish my worst enemy should undergo [self] quarantine because you will be so bored. In order not to be scared about the disease, I was also not watching television, and that made the boredom worse. my only comfort was the radio. I was tuning from one frequency to another just to listen to music and dance in the room in order deal with it [boredom]” [Female, 33 years old]*.

#### Anxiety

Having to wait for a period for the outcome of their test results also led to anxiety among participants. Due to insufficient COVID-19 testing centres at the beginning of the pandemic, test results for most suspected individuals were delayed. This caused some people under self-quarantine to become anxious, as they did not know what the outcome would be. One participant explained: *“For me, the fear was too much. The way they said people were dying in China and America and you are told you may be having the disease was very scary. To make matters worse, after I was told to self-quarantine, they did not visit me again. In my case, I left the room myself before they came to the house that I am free to go out. I left because my business was suffering” [Male, 39 years old]*.

## Discussion

In this study, we examined challenges associated with COVID-19-related self-quarantine among selected persons in the Eastern Region of Ghana. Three main challenges were identified: Socioeconomic, health-related, and psychological challenges. Socio-economic challenges participants reported included: lack of access to essential goods and services, loss of income, and poor housing conditions. The literature has shown that individuals in quarantine often experience difficulties in accessing essential goods and services such as food, with many facing shortages, price increases, and restricted access to stores [19]. This is often grave for people living in low- and middle-income settings where such goods and services are generally in short supply [20], which could have worsened during self-quarantine in the Eastern Region of Ghana. A plausible explanation could be that many people in low- and middle-income settings live in poverty and do not have the financial resources to stock up on supplies or order food and other necessities before undergoing quarantine or can order these goods and services online while under quarantine [20]. This, if not checked, could worsen the health conditions of persons placed under self-quarantine [21].

Participants also expressed concern about losing their income and source of livelihood because of undergoing self-quarantine. Research conducted by the International Labour Organisation [ILO] [22] showed that almost half of the global workforce, around 1.6 billion people, were at risk of losing their livelihoods due to COVID-19-related self-quarantine. Also, Bonaccorsi *et al*. [23] found that self-quarantine in Italy led to a decrease in income among self-employed individuals and those in non-essential sectors due to self-quarantine. Additionally, in Canada, individuals who were required to self-quarantine experienced a larger drop in income compared to those who were not required to self-quarantine, as they could not go about with their daily economic activities [24]. Similar findings have also been reported in China [25]. Hence, future self-quarantine regimes in the Eastern Region of Ghana could put in place economic loss-mitigating schemes for those quarantined to mitigate the economic impact such interventions may have on people. This is because a quarantine-related economic loss could lead to psychological stress, causing mental health problems, and may trigger long-lasting behaviour changes among individuals placed under self-quarantine [26].

Poor housing conditions, such as lack of potable water and toilet facilities, under which participants underwent self-quarantine, was another socio-economic challenge they encountered. This is in line with the literature. Research has shown that people living in overcrowded or poor housing conditions such as lack of access to private washrooms were more likely to face difficulties during COVID-19 related self-quarantine, even in the USA [27]. Similar challenges have also been reported in Nigeria, where individuals living in slums with poor sanitation were less likely to violate self-quarantine rules during the COVID-19 pandemic [28]. Consequently, future self-quarantine interventions in the Eastern Region of Ghana could ensure that housing conditions under which people are self-quarantined are conducive so as not to lead to worsened health outcomes among such individuals [29]. With reference to health-related challenges, participants mentioned sedentary lifestyle, non-supply of essential personal protective equipment such as face masks, development of oedema and weight gain as some of the challenges they faced. Participants were forced to practice sedentary lifestyle as self-quarantine altered their daily exercise routine and confined them to their rooms without much physical activity. This is consistent with research conducted in various parts of the world which reported a decline in all forms of physical activities due to COVID-19- related self-quarantine [30-32]. Sedentary lifestyle has been linked to poorer immune function, which may increase the risk of contracting other illnesses, aside COVID-19 [33]. With this in mind, individuals undergoing self-quarantine could be taught indoor exercising techniques to help them cope with sedentary lifestyle during times of isolation, to avoid the risks of developing non-communicable diseases [34].

The challenge of non-supply of essential medical items such as personal protective equipment for the self-quarantined exposed their caregivers to the COVID-19 virus in the Eastern Region of Ghana. Research elsewhere has shown that lack of nose masks was a barrier to compliance with self-quarantine measures among individuals in Hong Kong [35]. Thus, although isolated from the wider community, non-supply of basic PPEs could have served as an avenue for transmitting the virus from quarantined persons to caregivers and vice versa, should either party be infected with the virus. As such, basic health essentials, including face masks and hand sanitisers, could be provided to people who might undergo self-quarantine to effectively pandemic control interventions. Swollen foot was another health issue participant reported. The study revealed that the lack of movement made participants' feet to swell as they were placed under COVID-19-related self-quarantine. Research has shown that prolonged periods of inactivity and sitting can lead to the accumulation of fluid in the lower limbs, resulting in oedema as sitting or lying down for long periods can cause blood to pool in the lower extremities, leading to swelling [36-38]. Encouraging self-quarantined persons to perform exercises suited for confined spaces could help mitigate issues of swollen feet (oedema) during future self-quarantine interventions.

Moreover, some participants indicated that they gained extra weight during self-quarantine. According to research, the self-quarantine measures implemented during the COVID-19 pandemic resulted in a significant increase in weight gain among individuals as it negatively impacted physical activity levels [39]. Ammar and colleagues further reported an increase in consumption of high-calorie and processed foods, which may contribute to weight gain during the period of self-quarantine. Some researchers also argued that the COVID-19 pandemic resulted in a significant increase in stress levels among individuals who were placed under self-quarantine, which could have led to emotional eating and unhealthy food choices, contributing to weight gain and obesity [40]. Consequently, educating the quarantined on the right choice of diet as well as well as provision of counselling services to mitigate unhealthy emotional eating habits could address the negative consequences of future self-quarantine associated weight gain.

The third major challenge participants reported was psychological one. They explained that they faced psychological challenges such as loneliness, boredom and anxiety. Our findings suggested that some participants felt lonely as they were in isolation and wished to return to their normal lifestyles to mingle with people. This finding is consistent with a review conducted by [41], who found that during disease outbreaks such as the COVID-19, individuals who often undergo self-quarantine become lonely as their social networks temporarily disintegrate. Loneliness is often severe among self-quarantined adults and could lead to poorer mental health outcomes [42,43]. Meanwhile, research has shown that both loneliness and self-quarantine are independent risk factors for higher all-cause mortality [44], hence, efforts should be made to minimise loneliness among self-quarantined individuals in the future.

Our findings also indicated that some participants became bored during the period of self-quarantine as they could not perform their usual routines and had reduced social and physical contact with others. There was also a sense of isolation from the rest of the world, which was distressing to some participants made them [45]. Studies have shown that self-quarantine could lead to boredom and frustration due to limited activities that individuals can engage in [46]. It has been found that boredom could generate negative emotions, which may lead to depression, anxiety, loneliness, and lower levels of subjective wellbeing [46]. Thus, the provision of materials such as games that could mitigate boredom should be considered by health authorities in the Eastern Region for future self-quarantine interventions. Lastly, anxiety was another psychological challenge participants reported. This stemmed from the fact that they had to wait for a few days to receive their COVID-19 test results, which some of them never received. Same challenge was reported in China where suspected isolated COVID-19 persons suffered from anxiety due to uncertainty about their own health status [25]. Reviews conducted on the impact of self-quarantine during COVID-19 also reported anxiety as a main psychological challenge [47-49]. Anxiety, if not properly addressed, could lead to posttraumatic stress disorder (PTSD) [50]. Thus, while trying to contain the spread of an infectious disease such as COVID-19, efforts should be made to attend to the psychological needs of those quarantined.

### Lessons for future self-quarantine interventions

Future self-quarantine interventions in the Eastern Region of Ghana need to take into consideration socio-economic loss associated with the practice to find workable mechanisms that would restore the livelihoods of the affected, after self-quarantine. This could ensure that individuals are not economically worse off after self-quarantine, through no fault of theirs. In addition, free supply of essential medical supplies, such as PPEs to quarantined persons, should be considered. Moreover, education on basic exercises under quarantine conditions should be provided for individuals under self-quarantine to ensure that sedentary lifestyle, loneliness, and boredom do not creep in and negatively affect the health condition of the quarantined. Also, education on the right choice of meals under quarantine conditions should be given to individuals identified for self-quarantine to avoid unwanted weight gain during quarantine that could impact their health. Lastly, there is a need to attend to the psychological needs of those quarantined to minimise stress and anxiety and their associated health effects on those quarantined.

### Limitations of the study

Although efforts were made to ensure that our findings are trustworthy, we only interviewed thirty-five participants from the Lower Manya Krobo Municipality in the Eastern Region of Ghana. Consequently, our findings should be interpreted with caution, as generalising them to entire region or country might be out of context. However, the thoroughness of our processes gives enough credibility to our findings.

## Conclusion

COVID-19-related challenges that self-quarantined persons faced in the Eastern Region of Ghana were multifaceted, ranging from socio-economic, and health to psychological ones. Emergency preparedness for future pandemic control using self-quarantine as a tool should bring on board various stakeholders to ensure that the currently identified challenges are holistically addressed and do not recur.

### What is known about this topic

- *COVID-19 self-quarantine and weight gain related risk factors;*
- *Sports and exercise during self-quarantine*.

### What this study adds

- *Economic, physical and psychosocial challenges faced during COVID-19 self-quarantine in Ghana;*
- *Considerations for future self-quarantine interventions in Ghana*.
